# Artificial intelligence algorithms in orthopaedics: A narrative review of methods and clinical applications

**DOI:** 10.1002/jeo2.70549

**Published:** 2025-11-14

**Authors:** Jamie Rosen, Jemima Russell, Prerna Kartik, Martinique Vella‐Baldacchino

**Affiliations:** ^1^ Warwick Medical School University of Warwick Coventry UK; ^2^ MSk Lab, Department of Surgery and Cancer Imperial College London London UK; ^3^ Department of Trauma & Orthopaedics Kettering General Hospital Kettering UK

**Keywords:** algorithm, artificial intelligence, machine learning, orthopaedics, orthopaedic surgery

## Abstract

This narrative review evaluates the role of artificial intelligence (AI) algorithms in orthopaedic surgery and distinguishes itself by explaining how the main algorithmic approaches function and illustrating each with orthopaedic examples. Machine learning methods, including regression, classification and reinforcement learning, have been applied to fracture detection, prediction of revision risk and modelling of outcomes after arthroplasty and sports injury. Deep learning and convolutional neural networks have improved fracture classification, implant surveillance and segmentation of cartilage and meniscal tissue on magnetic resonance imaging. Neural networks such as FracNet and YOLO‐based systems demonstrate growing capability in trauma imaging. Natural language processing has automated the extraction of operative and registry data, while large language models are emerging for diagnostic support and education. Generative artificial intelligence (GAI) have produced synthetic musculoskeletal images to expand data sets. Computer vision and image processing underpin robotic‐assisted surgery and preoperative planning, and federated learning enables multicentre collaboration while protecting privacy. Each algorithm offers strengths in accuracy, efficiency or scalability, but also carries bias, transparency, computational cost and lack of external validation. This review explores how these algorithms are shaping orthopaedics, highlighting their benefits, limitations and challenges. Rigorous validation, transparent reporting and governance are essential for safe clinical use.

**Level of Evidence:**

N/A.

## Graphical abstract

Artificial intelligence (AI) algorithms are transforming orthopaedics through imaging and outcome prediction, but safe clinical translation requires robust validation, transparent reporting and effective governance.

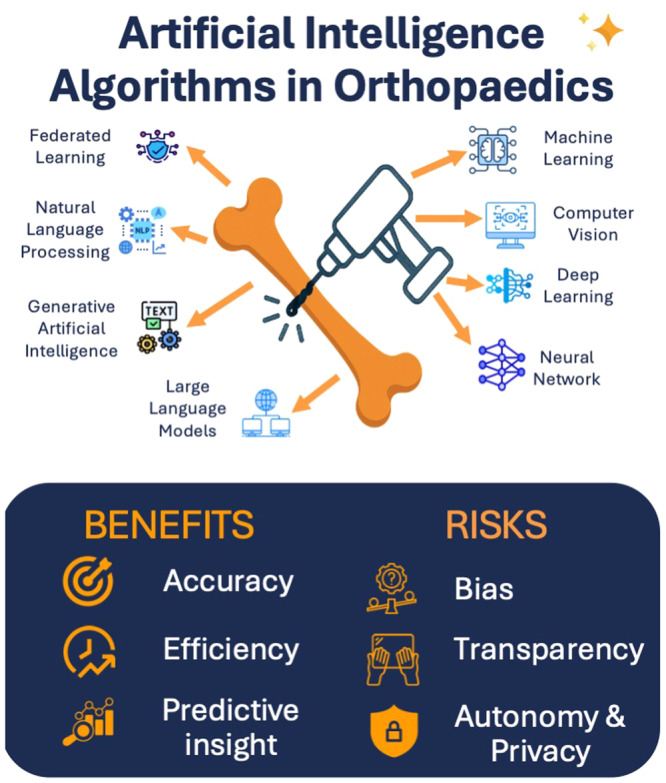

**Abbreviations**
AI: artificial intelligence
ANN: artificial neural network
AR: augmented reality
CNNs: convolutional neural networks
CT: computed tomography
CV: computer vision
DL: deep learning
DQN: Deep Q‐Networks
FL: federated learning
GAI: generative artificial intelligence
GANS: generative adversarial networks
IP: image processing
LLM: large language models
ML: machine learning
MR: mixed reality
MRI: magnetic resonance imaging
NLP: natural language processing
NN: neural network
ResNets: residual networks
RL: reinforcement learning
RNN: recurrent neural networks
SL: supervised learning
UL: unsupervised learning
VR: virtual reality

## INTRODUCTION

The origins of artificial intelligence (AI) date back to the mid‐20th century to Alan Turing, a pioneer in computer science who introduced the principle of machine learning (ML) [25]. Turing refers to a test known as the ‘Imitation Game’, whereby a person needs to determine whether they are communicating with a human or a machine based entirely on written conversation [96]. This paved the way for further study and development in the area of ML. A key development has been the emergence of deep learning (DL) architectures such as Residual Networks (ResNets), which have transformed medical imaging by enabling accurate feature extraction from complex data sets [34].

AI is a broad concept that refers to the capability for computer software to mimic human intelligence [45]. The term AI was formally introduced by Stanford University professor of computer science John McCarthy at Dartmouth in 1956, where he wanted to see if a machine could simulate human intelligence [8]. AI acts as an umbrella for many different subareas including machine learning (ML), deep learning (DL), neural networks (NN), natural language processing (NLP) and large language models (LLMs), computer vision (CV) and image processing (IP), federated learning (FL) and generative artificial intelligence (GAI) [53, 68]. Many of these approaches are already incorporated into orthopaedic and musculoskeletal surgery. For example, NNs are applied to fracture detection, NLP to operative notes and administrative tasks and GAI models to synthetic musculoskeletal imaging [5, 14, 105]. This review seeks to provide an overview of how these algorithms function and illustrate their applications to orthopaedic surgery, while also addressing their benefits, risks and future directions.

## METHODS

### Research methodology

This review was conducted as a narrative review. No protocol registration or PRISMA reporting framework was applied. A structured literature search was conducted in Ovid MEDLINE and Embase for studies published between 2021 and 2025. This timeframe was chosen to reflect recent advances in AI and the progress in clinical validation that has influenced orthopaedic practice. In addition, key journals in orthopaedics and related fields were manually screened to identify further relevant studies. To provide historical context, earlier influential works were also included. These included the history of AI, the introduction of convolutional neural networks (CNNs), the AlexNet architecture and landmark applications in clinical imaging. Such studies have provided the foundations of current AI methodologies in orthopaedics. Abstracts, preprints and animal studies were excluded.

The full search strategy is provided in the Supporting Information S1: Appendix. This included combinations of AI and algorithms with orthopaedic and related fields (trauma, fracture, arthroplasty, joint replacement), including subfields such as machine learning, deep learning and NLP. Full‐text studies in English focusing on algorithm functionality, clinical applications, benefits, risks and implementation challenges in orthopaedics were included. Both original research and review articles were included to ensure a broad overview of the field. All studies were imported into Rayyan: Systematic Review Management Platform. Title, abstract and full‐text screening were conducted independently and blindly by two reviewers. Included studies were then extracted into Microsoft Excel and organised thematically according to the type of AI technique. No formal critical appraisal was undertaken.

## RESULTS

### Key areas of AI

#### Machine learning (ML)

ML is a foundational subset of AI concerned with developing algorithms that analyse large data sets, perform specific tasks and improve performance through experience without explicit reprogramming [72]. It is often defined as a ‘computer programme that learns from experience with respect to some class of tasks T and performance measure P, if its performance at tasks in T, as measured by P, improves with experience E′ [62].

In orthopaedics, ML applications include automated fracture detection and classifications on radiographs, predicting revision risk following joint replacements and a patient's length of stay in hospital [12, 21, 51]. ML has also been applied to gait and motion analysis for injury and fall risk assessments, optimising rehabilitation strategies and predicting return to sport timelines for athletes [22, 78].

#### Deep learning (DL)

DL is a specialised subset of ML employing multilayered artificial neural networks (ANNs) to model complex, non‐linear relationships and automatically extract hierarchical features. DL processes unstructured data, with foundational research establishing the theoretical and practical basis for its current use in orthopaedic practice [24, 54].

DL has been applied to radiological imaging including the detection of fractures on X‐rays and for identifying spinal stenosis and degenerative changes on magnetic resonance imaging (MRIs) [4, 19]. Beyond fracture detection, it has been applied to pelvic fracture classification using the AO/OTA pelvic fracture classification scale highlighting how DL can support decision‐making in orthopaedic trauma [55]. DL models' ability to handle large amounts of complex data enables the segmentation of meniscal and cartilage tissue on MRIs, which can support arthroscopic assessment [13]. Recently, DL models have been used to predict implant loosening by analysing radiographs [47]. This highlights the potential of DL to improve long‐term arthroplasty surveillance.

#### Neural networks (NN)

NNs comprise interconnected input, hidden and output layers that iteratively transform data to identify complex patterns [87]. Orthopaedic NNs have demonstrated promising outcomes in detecting fractures on radiographs with limited data sets [4]. Orthopaedic NN FracNet combines supervised learning (SL) with feature fusion in order to improve fracture recognition accuracy [1].

CNNs are optimised for image analysis, capturing spatial hierarchies through their layered filters. The AlexNet architecture marked a turning point in CV, enabling widespread adoption of deep CNNs in healthcare [49]. CNNs have successfully automated the classification of distal radius fractures on radiographs, performing at the level of an orthopaedic surgeon [71, 75]. CNNs also support paediatric orthopaedic applications with models such as YOLOv8‐AM improving fracture detection in children. These examples highlight how CNNs are becoming increasingly capable of enhancing the speed and accuracy of radiological interpretation in orthopaedics [7].

#### Natural language processing (NLP) and large language models (LLM)

NLP enables computational systems to process, interpret and generate human language, structuring information from unstructured data sources as electronic health records, discharge summaries and consultation transcripts [100, 105]. Applications within orthopaedics include extracting key fields such as operative approach, fixation method and bearing surfaces from postoperative notes following arthroplasty surgery [101]. NLP has also been used to analyse large sets of musculoskeletal radiology reports to identify fracture trends, which reduces the need for clinicians to manually check promoting more efficient research [43].

LLMs build on NLP by using DL and transformer‐based architectures to generate contextually relevant, human‐like text [79]. They are trained on extensive volumes and varying types of data, including open‐access literature and other publicly available sources [63]. LLMs have shown the potential to support orthopaedics through the generation of educational content for both clinicians and patients [81]. They play a growing role in diagnostics and research by managing large data sets and integrating with other AI approaches to enhance accuracy [111]. Their autoregressive design enables next‐token prediction based on probabilistic modelling [106].

#### Computer vision (CV) and image processing (IP)

CV uses algorithms, often integrated with DL, to interpret and analyse visual data. IP is a CV subfield that employs techniques such as noise reduction, contrast enhancement and segmentation to optimise diagnostic images [23, 49]. CV has been applied to interpret pelvic x‐rays and detect fractures with high accuracy and automate AO‐OTA fracture classification [29]. This highlights the impact to both diagnostic and preoperative planning. IP techniques such as photon‐counting computed tomography (CT) with virtual monoenergetic reconstruction reduce metal artefacts around implants, providing clinicians with better visibility of bone and soft tissue structures [86]. IP is a key feature of the Mako surgical robot which uses a preoperative CT scan and three‐dimensional (3D) model reconstruction to create a 3D patient‐specific plan for surgery [27].

#### Federated learning (FL)

FL enables collaborative model training across institutions without transferring raw patient data, thereby preserving privacy and addressing governance concerns [94, 109]. FL has been explored in spine surgery, where it shows promise for robotic telesurgery, vertebral image segmentation, privacy‐preserving predictive modelling and enabling multicentre collaboration without sharing sensitive patient data [88].

#### Generative artificial intelligence (GAI)

GAI creates new content such as audio, code, images, text and videos based on learned data patterns [50]. GAI has demonstrated its ability to generate orthopaedic discharge documents more efficiently than clinicians whilst maintaining comparable writing quality [82]. GAI has also been used to generate synthetic radiographs and CT scans of spinal fractures [14, 20, 58]. These methods can expand training data sets and enhance diagnostic models without compromising patient confidentiality. However, outputs require rigorous expert validation before clinical application [14].

### Key AI approaches

#### Machine learning

ML models can be split into three main types of models: supervised, unsupervised and reinforcement learning. SL is a technique where labelled data sets are used to train algorithmic models in order to predict a certain outcome, such as the presence of a disease or tumour [104]. Unsupervised learning (UL) is where unlabelled data sets are used to train models [26]. Reinforcement learning (RL) is where the algorithms learn based on trial and error and receive feedback, which allows them to learn [3]. ML Models will learn using a combination of SL, UL and RL using both human input and large data sets to ensure that models can handle new tasks [33]. Table 1 provides an overview of the mechanisms of common ML algorithms, and Table 2 presents the associated benefits and risks of their application in orthopaedics.

**Table 1:** Overview of common machine learning algorithms and their mechanism.

Algorithm	Type	Mechanism
Regression	SL	Continuously predicts values based on input data by establishing relationships between variables such as *X* and *Y*.
Linear Lasso Random forest Gradient boosting		
Classification	SL	Assign data to specific categories by analysing their characteristics and features.
Logistic KNN SVM		
Naïve Bayes classifiers:	SL	Applies Bayes' mathematical theorem of probability to predict a data category and sort items into groups with the highest probabilities [3].
Decision trees		
K‐means clustering	SL	Separates data into groups that are most related or similar and assigns them accordingly.
Hierarchical clustering	UL	Creates a tree of different groups and iteratively grouping and assigning data based on similarity [69, 92].
Probabilistic clustering	UL	Assigns data to groups by estimating the likelihood of the data belonging to a particular group.
Q‐Learning	RL	Uses an agent (computer software) that learns by updating values in a table to establish which actions yield the best results.
DQN	RL	Works like the Q‐Learning algorithm but uses a NN instead of a table to establish which actions give the best results, so it can handle larger and more complex data sets.

Abbreviations: DQN, Deep Q‐Networks; KNN, K‐nearest neighbours; NN, neural network; RL, reinforcement learning; SL, supervised learning; SVM, support‐vector machines; UL, unsupervised learning.

**Table 2:** Summary of machine learning algorithms with associated benefits and risks in orthopaedic applications.

Algorithm	Type	Benefits	Risks
Regression:	SL	Quick to train, it is widely used in practice and is effective at predicting numerical values [85].	Cannot function with complex data or patterns and can be thrown off by unexpected data. Assumes a straight‐line relationship between variables [85].
Linear Lasso Random forest Gradient boosting			
Classification: Logistic KNN SVM	SL	Works well with different data types, can process complex patterns/relationships and is commonly implemented [85].	High computational cost, struggle interpreting complex models such as DL and can produce biased results if training data is biased [59, 85].
Naïve Bayes classifiers: Decision trees	SL	Simple to implement, does not require significant amounts of data to be trained and can handle different data types as input [15].	Can make inaccurate predictions if trained on incorrect data, struggles to compute new data and can assign incorrect values to this, and treats all data as separate even if there is a clear relationship [15].
K‐means clustering	UL	Simple to implement, it is fast and can compute relatively large data sets due to its low computational requirements; it is easy to interpret and is used widely across many fields [28, 38, 40].	Sensitive to initial values resulting in suboptimal grouping of data, a few unexpected values can skew results, and users can struggle to group data that forms nonspherical or irregular shapes [28, 38, 40].
Hierarchical clustering	UL	Reveals a clear hierarchical structure which is easy to interpret, and there is no preset number of clusters, so you can decide the number of groups later on making analysis of results easier [77, 110].	Large computational requirements, not scalable for very large data sets, and the outcome can vary based on parameters chosen, potentially leading to different cluster structures [56, 77, 110].
Probabilistic clustering	UL	Capable of analysing groups even if in an irregular shape and can handle data or values that are clear or have errors in [28].	Slow when working with large volumes of complex data, and results can be sensitive to initial values [28].
Q‐Learning	RL	It can function with limited initial data and is useful for uncertain situations [80].	Can overestimate values, leading to inaccurate outcomes and poor decision‐making, and can be slow when working with large and complex data sets [80].
DQN	RL	Useful in complex data analysis and recognising complex patterns [76].	Requires large data sets, requires significant computing power and training time, and can sometimes overestimate values, leading to inaccuracies [76].

Abbreviations: DL, deep learning; DQN, Deep Q‐Networks; KNN, K‐nearest neighbours; RL, reinforcement learning; SL, supervised learning; UL, unsupervised learning.

#### Neural networks

NN act as an umbrella for different types of NN models. These include but are not limited to CNNs, generative adversarial networks (GANs) and recurrent neural networks (RNNs). CNNs use the mathematical operation of convolution to recognise different patterns in images by analysing key features such as shape, colour or objects [36]. GANs are made up of two parts, with one part generating a fake image or other types of data and the other part determining if it is fake or not [2]. These two parts compete in order to make the best fake. RNNs process data and remember previous data they processed, which allows them to recognise complex patterns from sources such as text or speech [60]. Table 3 summarises the types of neural networks, their applications in orthopaedics and associated benefits and risks.

**Table 3:** Summary of types, applications in orthopaedics, benefits and risks of NNs.

Type of NN	Application in orthopaedics	Benefits	Risks
CNNs	Detection and classification of fractures, including distal radius, proximal humerus, hip, pelvis, femur and spine [39, 70]. Automated identification of hip and knee arthroplasty implants and prediction of prosthetic loosening [9, 30, 47].	Self learns what to look for when extracting, so it does not need to be told everything and is effective at pattern recognition in images and objects [6, 112].	Needs a lot of examples to be proficient and can struggle to analyse images different from what it has been trained on [6, 112].
GANs	Generating synthetic musculoskeletal radiographs to validate ML algorithms and train CAD systems [46, 74].	Can generate synthetic content which looks real, which can be useful for educational purposes, and content does not need to be labelled as GAN can determine labels [91, 98]. Protect patient privacy due to the synthetic nature of the images being generated [14, 46].	May keep generating the same content instead of new variations and can be hard to train as it can be unpredictable [91, 98].
RNNs	Gait analysis and fall prediction based on longitudinal balance monitoring [108]. Analysis of biometric and motion data in order to identify injury patterns in athletes [84].	Can handle data of varying lengths and sequences and can understand data presented in an order, making it ideal for handling text, speech or time‐based data [60, 99].	Requires significant computing power and time to train and has a poor memory, so struggles to remember data past a certain timeframe [60, 99].

Abbreviations: CAD, computer‐aided diagnosis; CNNs, convolutional neural networks; GANs, generative adversarial networks; ML, machine learning; NNs, neural networks; RNNs, recurrent neural networks.

### Benefits of AI in orthopaedics

The implementation of AI has already started to transform orthopaedic care. From imaging interpretation and preoperative planning to conducting administrative tasks, AI has been deployed across the surgical pathway. Recent literature highlights AI's potential to enhance diagnostic accuracy, alleviate clinician workload and promote the use of predictive analytics for earlier intervention [32, 57].

#### Improved diagnostic accuracy

AI has demonstrated diagnostic accuracy that in some orthopaedic imaging tasks exceeds or performs comparably to that of clinical experts. Performance in AI models is often measured using the ‘F1 score′. This metric is the harmonic mean of precision and recall and ranges from 0 (poor) to 1 (perfect) [73]. In paediatric distal radius fracture detection, CNNs outperformed experienced radiologists and orthopaedic surgeons. They achieved F1 scores of 0.90–0.93 compared with 0.77–0.86 for clinicians and area under the curves (AUCs) of 0.95–0.98 versus 0.94 for clinicians [42]. Similarly, in a study on hip fracture detection using anteroposterior pelvic radiographs, a DL ensemble achieved an F1 score of 0.942, surpassing orthopaedic surgeons (0.938) and radiologists (0.677). The model also reached a sensitivity of 0.97 and a specificity of 0.915 [11]. Whilst AI models do not consistently surpass orthopaedic surgeons in fracture detection, they often outperform nonorthopaedic clinicians [17]. This suggests there might be potential value of AI as a training tool for residents and other clinicians. A recent systematic review and meta‐analysis of hip fracture detection found AI accuracy to be inferior to musculoskeletal specialists, highlighting the need for further research and model training before clinical deployment [67].

#### Alleviates strain on clinicians and optimises resource use

Across the orthopaedic surgical pathway, AI reduces repetitive tasks that increase both clinical and administrative workloads. AI support improves fracture interpretation with a 29% reduction in missed fractures and 21% fewer false positives, without increasing reading time [10]. A systematic review and meta‐analysis of commercial fracture‐AI tools reported time savings of approximately 6–12 s per radiograph when AI was used to support a clinician [37]. Beyond imaging, NLP extracted key fields from postoperative total hip arthroplasty (THA) notes with 100% accuracy, enabling automated registry updates rather than manual entry [101]. ChatGPT also produced discharge documents for orthopaedic patients almost 10 times faster than physicians with comparable quality, highlighting AI's ability to reduce administrative workloads [82].

#### Predictive analytics and early intervention

AI's ability to analyse large complex sets of data and accurately predict patient outcomes enables earlier interventions, highlighting its advanced diagnostic capabilities [51]. ML applied to electronic health records improves fragility fracture prediction by providing a more personalised risk assessment than traditional tools like FRAX [35]. ML models predicting outcomes after total knee arthroplasty achieved AUCs of 0.79–0.84 accurately forecasting complications and discharge status [64]. Beyond clinical outcomes, models predicted the theatre duration of hip and knee arthroplasty with mean absolute errors of 11–13 min, enhancing theatre scheduling and resource allocation [52]. These advances demonstrate the potential of AI to promote earlier intervention and to optimise patient care in orthopaedics.

### Risks of AI in orthopaedics

While the adoption of AI in orthopaedics offers clear benefits, there are also significant risks that need to be addressed. These include bias arising from nonrepresentative training data sets, the ‘black box phenomenon’ that limits clinical insight into model decision‐making and significant privacy concerns related to the use of sensitive patient data.

#### Bias

There is a saying amongst computer scientists that an AI model is only as good as its data. If AI models are trained on nonrepresentative data sets, such as radiographs from only one patient demographic, this bias will be reflected in their outputs [16]. In orthopaedics, this can manifest as poorer accuracy in detecting paediatric fractures when algorithms are developed primarily from adult data sets. Systematic reviews have noted limited external validation and restricted reporting of demographic diversity [89]. Research has demonstrated that bias in hip and knee image segmentation can arise from imbalances in age, body size, ethnicity and sex. This reduces the generalisability of models across diverse patient populations [90]. Therefore, it is pivotal that representative, balanced data sets are used when training orthopaedic AI models to ensure equity of care.

#### Transparency (black box phenomenon)

The ‘black box phenomenon’ refers to the difficulty users face in understanding how AI models generate their outcomes [32]. This is particularly important for tasks such as fracture detection and outcome prediction, where surgeons must be able to evaluate why a model makes a specific decision. However, the black‐box nature of many models continues to hinder interpretability and limit clinical confidence [61]. A practical guide to AI implementation in orthopaedics has stressed that black‐box models present a major barrier to safe clinical translation, emphasising the need for explainability in high‐risk surgical settings [114]. Models predicting long‐term outcomes after THA demonstrate transparency by highlighting the clinical factors that most strongly influence predictions, making them easier for clinicians to understand [41]. Finally, recent systematic reviews recommend wider adoption of explainable AI methods to mitigate black‐box concerns and support safe human‐centred deployment [65].

#### Privacy and autonomy

The use of AI in orthopaedics requires the handling and processing of large amounts of sensitive patient data which creates a significant privacy risk [48]. Despite data being anonymised, breaches or association with external data sets can still enable reidentification [107]. Respect for patient autonomy also requires transparent disclosure and explicit consent when orthopaedic data is used for AI development. This is especially important given the opacity of many algorithms [44]. Decentralised approaches such as swarm learning have been successfully applied to fracture diagnosis, enabling multicentre collaboration while keeping data local and thereby reducing the risk of breaches [102].

## DISCUSSION

AI is reshaping orthopaedic workflows, with the most rapid progress in imaging interpretation, operative planning and outcome prediction. In fracture detection and arthroplasty imaging, models have demonstrated performance comparable to that of subspecialist clinicians. However, findings remain inconsistent, reflecting differences in data sets, fracture types and modelling approaches [29, 47, 57, 67]. ChatGPT‐4o recently showed strong binary discrimination in knee osteoarthritis radiographs (F1 = 0.88) but poor grading accuracy (AI [35% correct] vs. surgeons [89.6% correct]), highlighting inconsistencies in AI radiographic classification [113]. Progress will depend on the use and validation of representative orthopaedic training data. Recent reporting frameworks TRIPOD+AI, PROBAST+AI and DECIDE−AI can help ensure such studies are transparent, reproducible and clinically meaningful [18, 66, 97].

GAI and LLMs are beginning to show utility in orthopaedics, particularly for documentation, registry curation and patient‐facing education. Pilot studies suggest that LLMs can generate discharge summaries substantially faster than clinicians while maintaining comparable quality [82]. NLP has also been validated for extracting key operative details from arthroplasty notes with very high accuracy, supporting registry automation [101]. LLMs have further demonstrated value in improving the readability of orthopaedic patient education materials without compromising accuracy [81]. A recent evaluation of ChatGPT‐4o and DeepSeek R1 for ACL surgery patient education found both models were highly accurate (3.9/4) and consistent (4/4) on a 4‐point Likert scale. ChatGPT gave more comprehensive answers (4.0 vs. 3.2, *p* < 0.001), while DeepSeek provided clearer layperson‐focused responses (3.9 vs. 3.0, *p* < 0.001), illustrating variability in LLM outputs for patient education [31].

A recent systematic review found that most orthopaedic LLM studies report efficiency gains, but clinical performance remains highly variable, with diagnostic accuracy ranging from 55% to 93% [111]. While these tools may enhance workflow and patient communication, their integration into high‐stakes applications such as implant selection or intraoperative guidance will depend on explainability, transparency and close clinician oversight [41, 114].

Regulatory frameworks are now an important factor in how orthopaedic AI can be implemented. The EU AI Act (2024) introduces a risk‐based approach. It places obligations on high‐risk medical AI systems and on certain general‐purpose models. These measures are directly relevant to decision support, imaging and robotics [95]. Platforms that generate patient‐specific surgical plans demonstrate the clinical utility of AI, but they also highlight the need to clarify accountability at each stage of the surgical pathway [27]. Federated learning offers another development. It enables multi‐centre collaboration in spine and fracture research while preserving privacy and avoiding direct data sharing [88]. To strengthen reporting of diagnostic accuracy studies involving AI, the new STARD‐AI guideline provides items specific to ML applications [93].

Economic and workflow impact are important factors for AI adoption. ML models have been used to predict theatre duration, length of stay and implant survivorship, showing potential for resource optimisation [52]. In fracture detection, AI support reduces missed fractures and false positives without increasing reading time. Commercial tools have also shown modest time savings per radiograph, which may scale to service‐level efficiency gains [10, 37]. NLP has demonstrated high accuracy in extracting registry variables from arthroplasty notes, suggesting a role in reducing manual data entry [101]. Opportunities also exist in education and training, such as simulation and synthetic data sets, but these remain underevaluated in prospective orthopaedic settings. One study found ChatGpT‐4o achieved a mean score of 70.7% compared to candidates 58% on the Turkish orthopaedics and traumatology board exam. ChatGPT scored much higher in basic science (93% vs. 55%) but only slightly better in limb reconstruction (70% vs. 66%) [103]. LLMs appear capable of competitive exam‐level performance in certain domains but not consistently throughout orthopaedic subspecialties.

Recent evidence suggests that rigorous validation, transparent reporting and lifecycle governance are as important as algorithmic advances for safe integration of AI in orthopaedics. Collaboration between engineers and surgeons, evaluation against workflow‐relevant outcomes and postdeployment monitoring should become minimum standards. Equally important is preparing both surgeons and patients. Surgeons will need practical training in how to use and interpret these tools safely. Patients should receive clear explanations of how these tools function and how their data is handled. A recent study of 360 orthopaedic surgeons found that most expect AI to substantially impact practice within 5–10 years, predominantly as a complementary tool rather than a replacement [83]. Trust in AI will depend on validation, transparency and governance.

### Limitations

This review has several limitations. This review was restricted to English‐language studies published between 2021 and 2025. While selected seminal works published before this period were included to provide historical and methodological context, it remains possible that other relevant earlier studies were not captured. Language or publication bias may also have been introduced. Formal bias assessment tools were not used which limits evaluation of study quality. The rapidly evolving nature of AI means that some findings may become outdated. Finally, the search was limited to two databases, so additional relevant studies may not have been captured.

### Future research directions

Future research in the use of AI in orthopaedics needs to go beyond retrospective studies. There is a lack of solid prospective research that directly compares the performance of AI models with surgeons, especially in the areas of fracture detection and outcome prediction. As most of the studies included in this paper were conducted at single centres, their findings are unlikely to be representative of broader hospital settings or diverse patient populations. Integrating AI with trauma and implant registries could enhance long‐term tracking. Economic evaluations, such as the impact on theatre time or hospital stays, are rare but essential for judging real‐world value. Simulation and synthetic data sets also show promise for training, though they have not yet been properly tested in real clinical settings.

## CONCLUSION

AI is reshaping orthopaedics. It not only improves imaging interpretation and predictive modelling but also helps ease clinical and administrative burdens. While studies have shown better diagnostic accuracy and more efficient workflows, this progress is not without challenges. Bias remains a concern. Transparency is often limited, and stronger rules and oversight are needed for safe implementation. AI should be viewed as a tool to complement a surgeon and support decision‐making when integrated appropriately. With proper testing, responsible rollout and collaboration between experts, it has the potential to raise the standard of patient care in orthopaedics and beyond. Achieving this will require ongoing evaluation and shared accountability.

## AUTHOR CONTRIBUTIONS

Jamie Rosen, Jemima Russell, Prerna Kartik and Martinique Vella‐Baldacchino each made substantial contributions to this work, including study design, data collection, analysis and interpretation. Jamie Rosen drafted the initial manuscript, and all authors (Jamie Rosen, Jemima Russell, Prerna Kartik and Martinique Vella‐Baldacchino) were involved in revising the manuscript and gave final approval for the version to be published. All authors had access to all data in the study and take responsibility for the integrity of the data and the accuracy of the data analysis. The corresponding author attests that all listed authors meet authorship criteria and that no others meeting the criteria have been omitted.

## CONFLICT OF INTEREST STATEMENT

The authors declare no conflict of interest.

## ETHICS STATEMENT

The authors have nothing to report.

## Supporting information

Appendix.
