# Supplementary material for: Artificial intelligence algorithms in orthopaedics: A narrative review of methods and clinical applications
Source: J Exp Orthop. 2025 Nov 14;12(4):e70549. doi: 10.1002/jeo2.70549 (PMC12616508; doi:10.1002/jeo2.70549)
Supplement: Supplementary file 1 — Appendix. [file JEO2-12-e70549-s001.docx]

# Search String

1 exp Orthopedics/ or exp Orthopedic Procedures/ or exp Fractures, Bone/ or exp Arthroplasty, Replacement, Knee/ or exp Arthroplasty, Replacement, Hip/ or exp Spine/

2 (orthopaedic* or orthopedic* or fracture* or arthroplasty or implant* or prosthes* or spine or hip or knee or shoulder or "rotator cuff" or meniscus or "anterior cruciate ligament" or ACL or cartilage or trauma).ti,ab,kw.

3 exp Artificial Intelligence/ or exp Machine Learning/ or exp Deep Learning/ or exp Neural Networks, Computer/ or exp Natural Language Processing/

4 (machine learning or deep learning or "neural network*" or CNN or "convolutional neural network*" or "large language model*" or LLM or natural language processing or NLP or computer vision or generative AI or federated learning or swarm learning).ti,ab,kw.

5 1 or 2

6 3 or 4

7 5 and 6

8 limit 7 to english language

9 limit 8 to full text

10 limit 9 to yr="2021 - 2025"

11 limit 10 to "remove preprint records"
